# Supplementary material for: Calcium dynamics regulating the timing of decision-making in C. elegans
Source: eLife. 2017 May 23;6:e21629. doi: 10.7554/eLife.21629 (PMC5441874; doi:10.7554/eLife.21629)
Supplement: Supplementary file 2. — DOI: http://dx.doi.org/10.7554/eLife.21629.028 [file elife-21629-supp2.docx]

**Supplementary File 2. Plasmids used in this study.**

| **Plasmid name** | **Promoter** | **cDNA** | **Vector backbone** | **Cloning strategy** |
| --- | --- | --- | --- | --- |
| pKAW70 | *str-1* (1,083 bp upstream sequence of *str-1* gene for AWB expression) | ChR2  (C128S) | pPD96.52 | *str-1* promoter was PCR amplified from N2 genome and inserted into HindIII and BamHI sites of pCS86 (ChR2(C128S)::YFP, a gift from A. Gottschalk). |
| pKDK331 | *myo-3* (2,377 bp upstream sequence of *myo-3* gene for body wall muscle expression) | NLS-mRFP | pPD49.26 | *myo-3* promoter from pPD115.44 (a gift from A. Fire) was inserted into HindIII and XbaI site of pHK210-NLS (NLS-mRFP in pPD49.26, a gift from H. Kagoshima). |
| pKFU177 | *sra-6* (4,005 bp upstream sequence of *sra-6* gene for ASH expression) | mCherry | pPD49.26 | *sra-6* promoter was amplified from N2 genome by PCR and inserted into HindIII and XbaI sites of pMIY69. |
| pMIY69 | n/a | mCherry | pPD49.26 | mCherry (Clontech) was PCR amplified and inserted into NheI (vector) / SpeI (insert) and EcoRV sites of pPD49.26 (a gift from A. Fire). |
| pMIY88 | *str-1* (same with pKAW70) | mCherry | pPD49.26 | *str-1* promoter was inserted into SphI and BamHI sites of pMIY69. |
| pYFU53 | *str-1* (same with pKAW70) | GCaMP3 | pPD49.26 | GCaMP3 (AddGene) was PCR amplified and inserted into NheI (vector) / SpeI (insert) and EcoRV sites of pPD49.26. *str-1* promoter was inserted into SphI and BamHI sites of the plasmid. |
| pYFU77 | *srd-23* (3,024 bp upstream sequence of *srd-23* gene for AWB expression) | Arch | pPD49.26 | *srd-23* promoter was PCR amplified from N2 genome and inserted into HindIII and BamHI sites of pPD49.26. Arch was amplified from FCK-Arch-GFP (AddGene) by PCR, and inserted into KpnI and SacI sites of the plasmid. |
| pYFU107 | *sra-6* (same with pKFU177) | GCaMP3 | pPD49.26 | GCaMP3 (AddGene) was PCR amplified and inserted into NheI (vector) / SpeI (insert) and EcoRV sites of pPD49.26. *sra-6* promoter from pKFU177 was inserted into XbaI site of the plasmid. |
| pYFU110 | *sra-6* (same with pKFU177) | Arch | pPD49.26 | Arch was inserted into BamHI and SacI sites of pKFU177. |
