# Supplementary material for: Calcium dynamics regulating the timing of decision-making in C. elegans
Source: eLife. 2017 May 23;6:e21629. doi: 10.7554/eLife.21629 (PMC5441874; doi:10.7554/eLife.21629)
Supplement: Supplementary file 3. — DOI: http://dx.doi.org/10.7554/eLife.21629.029 [file elife-21629-supp3.docx]

**Supplementary File 3. Strains used in this study.**

| **Fig. no.** | **strain name** | **genotype** | **DNA injected** |
| --- | --- | --- | --- |
| 3C | KDK23009 | *N2;oskEx23009* | 80 ng/μl of *str-1p:GCaMP3* (pYFU53)  20 ng/μl of *str-1p:mCherry* (pMIY88) |
| 3C, 4B,  1-S1E,  4-S1, 4-S2 | KDK23031 | *N2;oskEx23031* | 50 ng/μl of *str-1p:GCaMP3* (pYFU53)  20 ng/μl of *str-1p:mCherry* (pMIY88)  30 ng/μl of PvuII-cut N2 genomic DNA as a carrier |
| 3C | KDK23034 | *N2;oskEx23034* | 50 ng/μl of *str-1p:GCaMP3* (pYFU53)  20 ng/μl of *str-1p:mCherry* (pMIY88)  30 ng/μl of PvuII-cut N2 genomic DNA as a carrier |
| 3C, 4B  6A, 6C  1-S1E,  4-S1, 4-S2 | KDK52204 | *N2;oskEx52204* | 50 ng/μl of *str-1p:GCaMP3* (pYFU53)  10 ng/μl of *str-1p:mCherry* (pMIY88)  30 ng/μl of PvuII-cut N2 genomic DNA as a carrier  10 ng/μl of *lin-44p::GFP* (a gift from M. Koga) |
| 3D | KDK41014  KDK41021 | *N2;oskEx41014*  *N2;oskEx41021* | 100 ng/μl of *str-1p:ChR2(C128S)::YFP* (pKAW70)  2 ng/μl of *myo-3p::NLS::mRFP* (pKDK331) |
| 3E, 4A, 7B, 7C, 7D, 4-S3, 7-S1 | KDK70034 | *N2;oskEx70034* | 20 ng/µl of *sra-6p::GCaMP3* (pYFU107) (pYFU107)  20 ng/µl of *sra-6p::mCherry* (pKFU177) (pKFU177)  10 ng/µl of *lin-44p::GFP*  50 ng/µl of PvuII-cut N2 genomic DNA as a carrier |
| 3E, 4A, 4-S3 | KDK70231  KDK70239  KDK70265 | *N2;oskEx70231*  *N2;oskEx70239*  *N2;oskEx70265* | 50 ng/µl of *sra-6p::GCaMP3* (pYFU107)  40 ng/µl of *sra-6p::mCherry* (pKFU177)  10 ng/µl of *lin-44p::GFP*  10 ng/µl of PvuII-cut N2 genomic DNA as a carrier |
| 3F | KDK42040 | *N2;oskEx42040* | 250 ng/µl of *sra-6p::Arch::GFP* (pYFU110)  2 ng/µl of *myo-3p::NLS-mRFP* (pKDK331)  5 ng/µl of PvuII-cut N2 genomic DNA as a carrier |
| 3F | KDK42096 | *N2;oskEx42096* | 50 ng/µl of *sra-6p::Arch::GFP* (pYFU110)  2 ng/µl of *myo-3p::NLS-mRFP* (pKDK331)  50 ng/µl of PvuII-cut N2 genomic DNA as a carrier |
| 4C | KDK41837  KDK41838 | *N2;oskEx41837*  *N2;oskEx41838* | 100 ng/µl of *srd-23p::Arch::GFP* (pYFU77)  2 ng/µl of *myo-3p::NLS-mRFP* (pKDK331) |
| 6A | KDK53638  KDK53643 | *unc-13(e51); oskEx52204*  *unc-31(e928);oskEx52204* | Please refer to *N2;oskEx52204* |
| 6B, 6-S1A | KDK70281  KDK70288 | *odr-3(n2150); oskEx23031*  *odr-3(n2150); oskEx52204* | Please refer to *N2;oskEx23031* and *N2;oskEx52204* |
| 7A, 7C | KDK70319  KDK70323  KDK70328  KDK70331 | *egl-19(n582); oskEx52204*  *unc-2(e55); oskEx52204*  *itr-1(sa73); oskEx52204*  *unc-68(e540); oskEx52204* | Please refer to *N2;oskEx52204* |
| 7B,  7C | KDK70325  KDK70343  KDK70330  KDK70333 | *unc-2(e55); oskEx70034*  *cca-1(ad1650);oskEx70034*  *itr-1(sa73); oskEx70034*  *unc-68(e540); oskEx70034* | Please refer to *N2;oskEx70034* |
| 6-S1A | KDK70306 KDK70318 | *odr-3(n1605); oskEx52204*  *odr-3(n1605); oskEx23031* | Please refer to *N2;oskEx23031* and *N2;oskEx52204* |
| 6-S1B | KDK70312  KDK70308 | *odr-3(n2150); oskEx70034*  *odr-3(n1605); oskEx70034* | Please refer to *N2;oskEx70034* |
| 7-S1 | KDK70352 | *unc-13(e51);oskEx70034* | Please refer to *N2;oskEx70034* |
